# Anti-Depressive Effectiveness of Baicalin In Vitro and In Vivo

**DOI:** 10.3390/molecules24020326

**Published:** 2019-01-17

**Authors:** Li Liu, Yu Dong, Xin Shan, Lin Li, Baomei Xia, Hanqing Wang

**Affiliations:** 1 College of Pharmacy, Guangdong Medical University, Dongguan 523808, China; liuli-44@163.com; 2 College of Pharmacy, Nanjing University of Chinese Medicine, Nanjing 210023, China; dongyu@njucm.edu.cn (Y.D.); shanxinqq@126.com (X.S.); astonishlilin@126.com (L.L.); 3 Faculty of Rehabilitation Science, Nanjing Normal University of Special Education, Nanjing 210023, China; babaysummer@163.com; 4 College of Pharmacy, Ningxia Medical University, Ningxia 750004, China

**Keywords:** baicalin, antidepressant, chronic unpredictable mild stress, inflammation, HMGB1/TLR4/NF-κB

## Abstract

Baicalin (BA), a major polyphenol compound isolated from the extracts of *Scutellaria radix*, has been previously reported to ameliorate depressive-like behaviors in mice with chronic unpredictable mild stress (CUMS). However, its underlying antidepressant mechanisms remain unclear. This study was designed to confirm the antidepressant-like effects of BA on CUMS induced behavioral abnormalities in mice, and sought to explore the pharmacological mechanisms in vivo and in vitro. The CUMS procedure was carried out to induce depression in mice. Afterwards, the tail suspension test (TST), forced swim test (FST), and open field test (OFT) were performed within 24 h, then sucrose preference test (SPT) was conducted. Additionally, PC12 cells were pretreated with BA for 2 h, then further stimulated with corticosterone for 24 h. The levels of Interleukin-1β (IL-1β), IL-6 and Tumor Necrosis Factor-α (TNF-α) in serum, hippocampus homogenate and cell culture medium were determined using the enzyme-linked immunosorbent assay (ELISA) method. The protein expressions of inhibition of high mobility group box 1 protein (HMGB1)/Toll-like receptor 4 (TLR4)/nuclear factor kappa B (NF-κB) pathways in hippocampus and PC12 cells were detected. Our results showed that CUMS-treated mice presented notable depressive-like symptoms, such as decreased sucrose consumption, increased FST and TST immobility time. While BA (25, 50 mg/kg) significantly attenuated these changes. Besides, BA treatment considerably inhibited inflammatory cytokinesl (IL-1β, IL-6, TNF-α) levels in serum, hippocampus homogenate and cell culture medium. Western blot analysis indicated that BA inhibited the expressions of HMGB1, TLR4, and p-NF-κBp65 both in vivo and in vitro. In conclusion, the present study confirmed that BA possessed efficient antidepressant effects on depression, which was possibly related to the inhibition of HMGB1/TLR4/NF-κB pathways.

## 1. Introduction

As one of the most common psychiatric disorders, depression is clinically featured by a chronic depressed mood, worthlessness feeling, cognitive impairment, fatigue, sleeplessness and even suicidal tendencies [1,2]. According to the World Health Organization, there are almost 350 million depressive patients in all ages around the world. Despite several decades of clinical effort, the underlying mechanism still remains poorly defined. Numerous evidence have found that the pathophysiology symptoms of depression contains cell proliferation dysregulation, neuroplasticity alteration, and abnormal inflammatory cytokine secretion [3,4]. The chronic unpredictable mild stress (CUMS) model of depression is widely used to mimic depressive behavior in the murine model and has proven predictive validity in a number of previous investigations [5]. CUMS is a chronic stress accounting for biochemical and behavioral derangements, which are related to endogenous depressions and neuropsychiatric disorders [6].

The cellular stressor and inflammation caused by CUMS can promote the generation of danger-associated molecular pattern (DAMP) molecules including high mobility group box 1 protein (HMGB1) [7,8]. DAMPs assemble with the receptors such as TLR and then lead to recruitment of the inflammasome [9]. The binding of HMGB1 to TLR4 triggers a cascade of proinflammatory modulators, such as interleukin-1beta (IL-1β) and tumor necrosis factor alpha (TNF-α). Intriguingly, The HMGB1/TLR4 complex further induced more HGMB1 secretion, which consequently augmented the inflammatory process. Previous research displayed that HMGB1 signaling was implicated in immune-associated disorders and neurological diseases [10]. HMGB1 drives depressive-like behavior attributed to chronic stress. It was proposed that the elevated levels of peripheral system and central nervous system HMGB1 protein were observed in mice with depressive symptoms [11]. Therefore, the inhibition of inflammatory reactions activated by the HMGB1 signaling pathway might provide effective pharmacological intervention on depressive behavior. 

Baicalin, a flavonoid compound extracted and purified from the dry roots of *Scutellaria baicalensis* Georgi (Huangqin), has several biological properties including antioxidant, anti-inflammatory and neuro-protective actions [12,13]. Several studies indicated the antidepressant-like effects of BA in a murine CUMS depression model [14,15]. However, its underlying mechanisms are complex and remain to be further explored. Therefore, the present investigation was to estimate whether BA treatment possessed the antidepressant-like effects on CUMS induced depression by inhibiting inflammation through down-regulation of HMGB1/TLR4/NF-κB signaling pathway.

## 2. Results

### 2.1. BA Improves CUMS Induced Depression-Related Behaviors

#### 2.1.1. Sucrose Consumption

**Figure 1:**
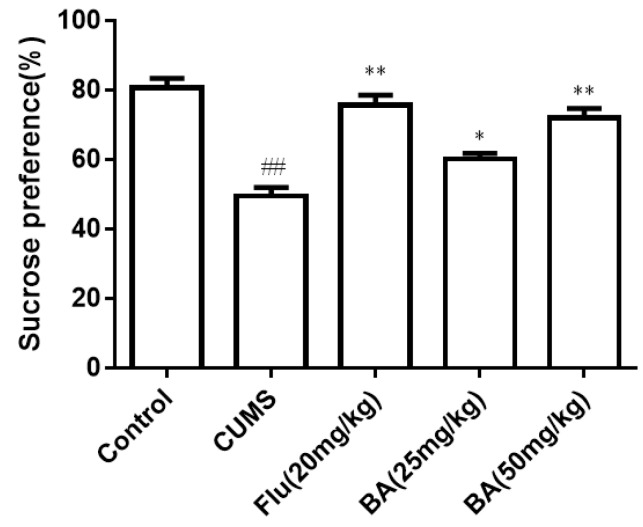
Effects of baicalin (BA) on Sucrose consumption. Data were expressed as mean ± SEM (*n* = 12). ## *p* < 0.01 vs. the control; ** *p* < 0.01, * *p* < 0.05 vs. the CUMS group.

The results of the sucrose preference (SPT) are illustrated in Figure 1. The sucrose intake was significantly reduced in induced CUMS group compared with that of the control group. On the contrary, the sucrose consumption in mice treated with BA (50 mg/kg) and fluoxetine (Flu, 20 mg/kg) was obviously increased compared with that in the CUMS group (*p* < 0.01). The efficiencies of BA (50 mg/kg) and Flu (20 mg/kg) were much stronger than that in the BA (25 mg/kg) group (*p* < 0.05). The data confirmed that BA could improve the sucrose preference in CUMS mice (F (4, 55) = 25.69, *p* < 0.01).

#### 2.1.2. The Effects of BA on Tail Suspension Test (TST) and Forced Swim Test (FST) 

**Figure 2:**
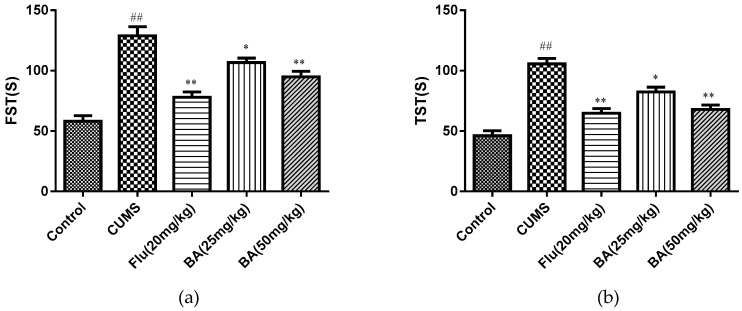
Effects of BA on (**a**) the tail suspension test (TST) and (**b**) the forced swim test (FST). Data were expressed as mean ± SEM (*n* = 12). ## *p* < 0.01 vs. the control; * *p* < 0.05 and **p < 0.01 vs. the CUMS group.

TST and FST are the most commonly used behavioral tests for evaluating antidepressants. As depicted in Figure 2, CUMS-treated mice caused increases in immobile durations for the TST and FST tests when compared with those in the control group. By contrast, BA (50 mg/kg) and Flu (20 mg/kg) treatment significantly decreased the CUMS induced elevation of immobility time in both TST (F (4, 55) = 29.99, *p* < 0.01) and FST ((F (4, 55) = 27.75, *p* < 0.01), while the treatment with BA (25 mg/kg) also reduced the immobility duration in both TST and FST tests (*p* < 0.05). 

#### 2.1.3. Effects of BA in the Open Field Test (OFT)

**Figure 3:**
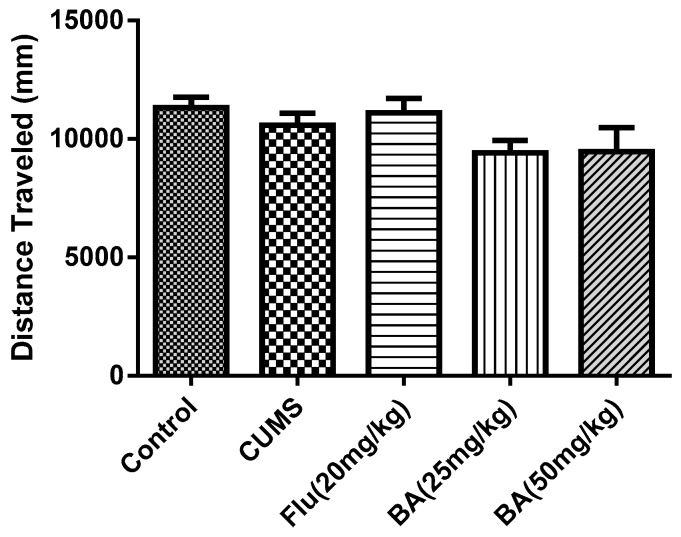
Effects of BA in the open field test (OFT). Data were expressed as mean ± SEM (*n* = 12).

It was found that the administrations of BA and Flu did not change either the locomotor activity (distance traveled) (Figure 3, F (4, 55) = 1.873, *p* = 0.128). This indicated that the reduction of immobility induced by acute BA treatment in the FST and TST was not due to locomotor hyperactivity.

### 2.2. Effects of BA on Pro-Inflammatory Cytokines in Mice

**Figure 4:**
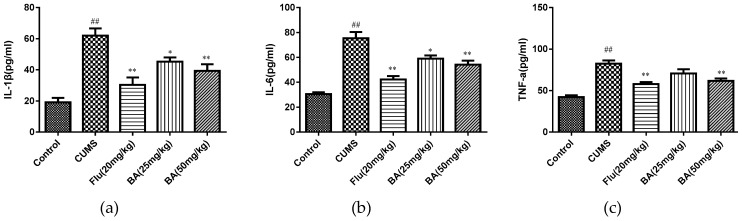
Effects of BA on proinflammatory cytokines (**a**) IL-1β, (**b**) IL-6, (**c**) TNF-α in serum. Data were expressed as mean ± SEM (*n* = 6). ## *p* < 0.01 vs. the control; ** *p* < 0.01, * *p* < 0.05 vs. the chronic unpredictable mild stress (CUMS) group.

**Figure 5:**
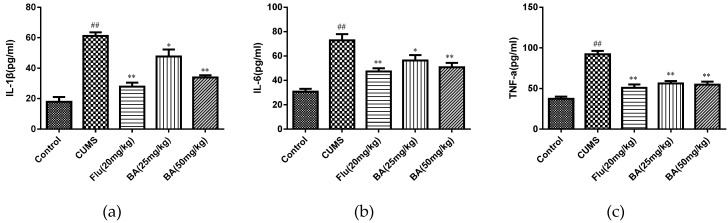
Effects of BA on proinflammatory cytokines (**a**) IL-1β, (**b**) IL-6, (**c**) TNF-α in the hippocampus tissues. Data were expressed as mean ± SEM (*n* = 6). ## *p* < 0.01 vs. the control; ** *p* < 0.01, * *p*< 0.05 vs. the CUMS group.

To evaluate the anti-inflammatory effects of BA, the levels of proinflammatory cytokines in the serum and hippocampus were determined by ELISA kits. As shown in Figure 4 and Figure 5, CUMS was a stimulator of proinflammatory cytokines including IL-1β, IL-6, and TNF-α in both serum and hippocampus (*p* < 0.01). Whereas the treatments with BA (50 mg/kg) and Flu (20 mg/kg) strongly decreased the serum concentrations of IL-6, IL-1β, and TNF-α compared with those in CUMS group (F (4, 25) = 28.01, *p* < 0.01 for IL-6; F (4, 25) = 12.47, *p* < 0.01 for IL-1β; F (4, 25) = 26.12, *p* < 0.01 for TNF-α).

Besides, the reductions of IL-1β, IL-6 cytokine concentrations in hippocampus were observed in BA (50 mg/kg) and Flu (20 mg/kg) groups (*p* < 0.01). The BA (25, 50 mg/kg) and Flu (20 mg/kg) treated group also suppressed the hippocampal generation of TNF-α (Figure 5, F (4, 25) = 18.14, *p* < 0.01 for IL-6; F (4, 25) = 42.1, *p* < 0.01 for IL-1β; F (4, 25) = 32.41, *p* < 0.01 for TNF-α). These analytical data proved that BA could inhibit the productions of inflammatory cytokines in both serum and hippocampus.

### 2.3. Effects of BA on HMGB1/TLR4/NF-κB Pathway in Mice

**Figure 6:**
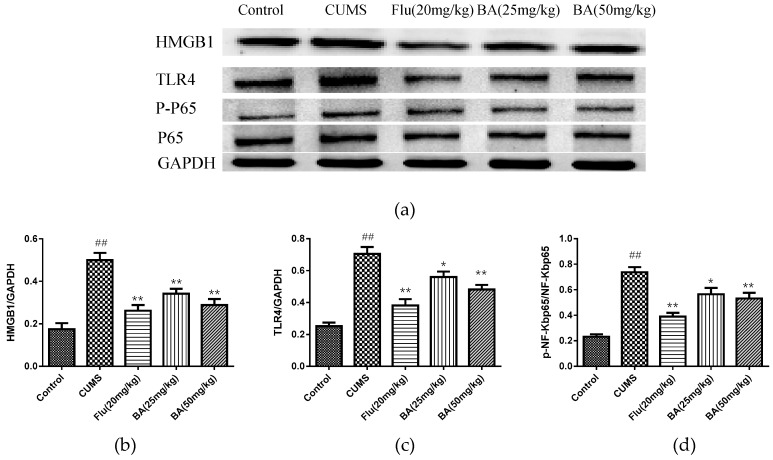
Effects of BA on (**a**,**b**) HMGB1/ (**a**,**c**) TLR4/ (**a**,**d**) NF-κB signaling pathway in mice (*n* = 3). Data were expressed as mean ± SEM (*n* = 6). ## *p* < 0.01 vs. the control; ** *p* < 0.01, * *p* < 0.05 vs. the CUMS group.

In order to identify the intracellular mechanism responsible for the antidepressant effect of BA in CUMS induced depression, the protein expressions of BA on HMGB1, TLR4, p-NF-κB, NF-κB in the hippocampus were investigated. As revealed in Figure 6, the up-regulated expressions of HMGB1, TLR4, and p-NF-κBp65 were observed in the hippocampal tissues of CUMS-exposed mice (*p* < 0.01). Nevertheless, BA (25, 50 mg/kg) and Flu (20 mg/kg) administrations dramatically down-regulated the HMGB1 expression (F (4, 25) = 18.27, *p* < 0.01). Moreover, the expressions of TLR4 and p-NF-κB were evidently suppressed with the treatments of BA (50 mg/kg) and Flu (20 mg/kg) (*p* < 0.01), which were more potent than those in BA (25 mg/kg) group (F (4, 25) = 25.48, *p* < 0.01 for TLR4; F (4, 25) = 24.7, *p* < 0.01 for p-NF-κB). 

### 2.4. Effects of BA on Cell Viability in PC12 Cells

**Figure 7:**
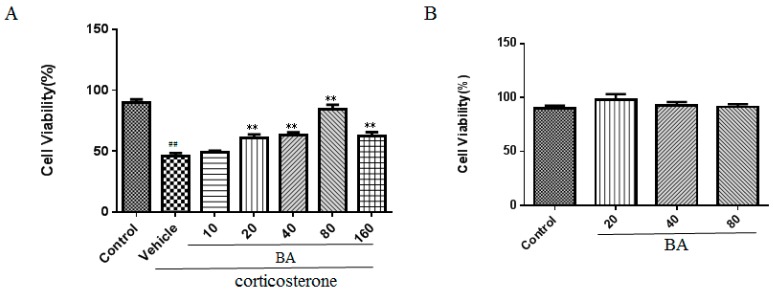
Effects of BA on cell viability in PC12 cells. (**A**) in the presence of corticosterone, (**B**) in the absence of corticosterone. Data were expressed as mean ± SEM (*n* = 6). ## *p* < 0.01 vs. the control; ** *p* < 0.01 vs. the corticosterone group.

Next, we examined the effect of BA on cell viability in vitro using the MTT method. As shown in Figure 7A, the results demonstrated that the corticosterone challenge obviously reduced the cell viability of PC12 cells as compared with that in the control group (F (6, 35) = 36.68, *p* < 0.01). Whereas the cell viability of PC12 cells was recovered by BA (20, 40, 80 μM) in concentration dependent manners as shown in our results (*p* < 0.01). It was noteworthy that BA (160 μM) decreased the cell viability possibly due to the cellular toxicity. Thus, we chose BA at the concentration of (20, 40, 80 μM) in the following test.

In order to investigate the cellular toxicity of BA, we performed a MTT assay. As illustrated in Figure 7B, no obvious significance among different concentrations of the BA (20, 40, 80 μM) groups and control group (F (3, 20) = 1.039, *p* = 0.3968) were found. It was indicated that BA did not notably affect the cell viability of PC12 cells and the protective effect of BA on corticosterone stimulation was not due to the cell toxicity. 

### 2.5. Effects of BA on Proinflammatory Cytokines in PC12 Cells 

**Figure 8:**
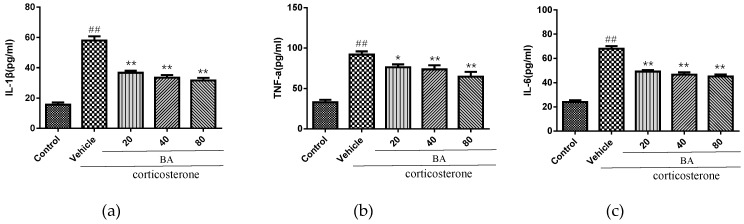
Effects of BA on proinflammatory cytokines (**a**) IL-1β, (**b**) TNF-α, (**c**) IL-6 in cell culture medium of corticosterone-induced PC12 cells (*n* = 6). Data were expressed as mean ± SEM (*n* = 6). ## *p* < 0.01 vs. the control; ** *p* < 0.01, * *p* < 0.05 vs. the corticosterone group.

We further studied the effect on the levels of inflammatory cytokines IL-1β, IL-6 and TNF-α in vitro. As shown in Figure 8, the corticosterone challenge caused the overproduction of IL-1β, IL-6 and TNF-α in cell culture medium (*p* < 0.01). Nevertheless, the treatments with BA (20, 40, 80 μM) strongly decreased the concentrations of IL-1β, IL-6 and TNF-α when compared with the corticosterone group (*p* < 0.01). Our ELISA data confirmed that BA was capable of suppressing the abundant secretion of inflammatory mediators in vitro.

### 2.6. Effects of BA on HMGB1/TLR4/NF-κB Pathway in PC12

**Figure 9:**
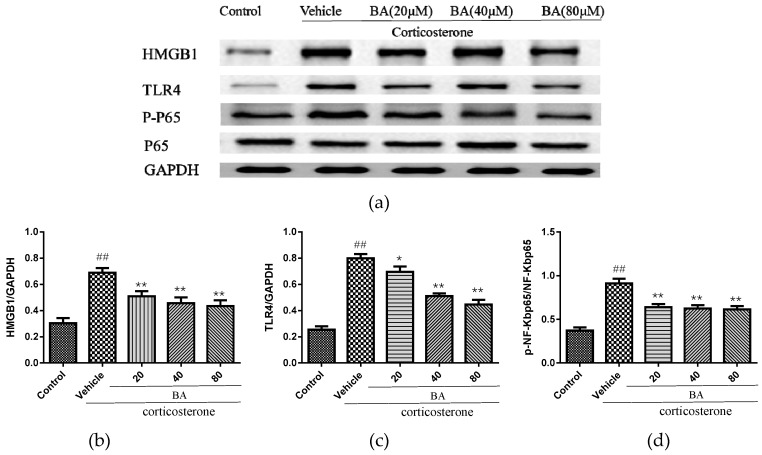
Effects of BA on (**a**,**b**) HMGB1/ (**a**,**c**) TLR4/ (**a**,**d**) NF-κB signaling pathway in PC12 cells. Data were expressed as mean ± SEM (*n* = 3). ## *p* < 0.01 vs. the control; * *p* < 0.05 and ** *p* < 0.01 vs. the corticosterone group.

In order to explore the underlying etiology associated with the protective effect of BA in corticosterone-induced PC12 cells, the protein levels of HMGB1, TLR4, p-NF-κB, NF-κB were detected. As revealed in Figure 9, the excessive expressions of HMGB1, TLR4 and p-NF-κBp65 were observed in the corticosterone group. While BA (20, 40, 80 μM) administrations dramatically decreased the levels of HMGB1, p-NF-κB levels (F (4, 25) = 11.87 *p* < 0.01 for HMGB1; F (4, 25) = 22.85, *p* < 0.01 for p-NF-κB). The treatments with BA (40, 80) also reduced the levels of TLR4 (*p* < 0.01), which were more efficient than that in the BA (20 μM) group (F (4, 25) = 45.15). The immunoblot results suggested that BA might attenuate the HMGB1/TLR4/NF-κB pathway in corticosterone-induced PC12 cells. 

## 3. Discussion

In this work, we aimed to investigate the antidepressant-like effect of BA in CUMS mice and its possible underlying mechanisms. Growing evidence suggested that chronic stress played a critical role in the pathophysiology of depression. In the present study, the CUMS depression model was established through stimulating animals with chronic randomizing different low-intensity stress factors to induce depressive status in the murine model. The model is similar with the clinical symptoms of depressive patients, and is widely used in screening anti-depressant drugs [16,17]. The results of pharmacological experiments indicated that BA significantly improved the CUMS-treated depressive-like behaviors in mice. The occurrence of depression is a multi-factor complex process [14]. It is well known that patients with depression exhibit higher levels of pro-inflammatory cytokines [18]. Thus, the anti-depressive activity of BA may be closely associated with its neuroprotective and anti-inflammatory effects [19].

Behavioral tests are important and widely performed to evaluate whether the drug possess anti-depressant activity. The reduction of partiality for sugar is implemented as an indicator for appetite lack [20]. In our work, the mice in the CUMS model group significantly suppressed the sucrose preference. BA treatment could significantly increase the sugar consumption in CUMS mice. Besides, OFT, TST and FST are also commonly used to evaluate the anti-depressive activity because of their high predictive validity [21]. BA administration reduced the immobility time in FST and TST, and restored sucrose consumption in SPT. The behavioral tests demonstrated that regular daily administration of BA could effectively ameliorate behavioral alterations in mice subjected to CUMS procedure. 

According to the cytokine hypothesis, depression may be triggered by psychosocial stressors and internal stressors through inflammatory processes [22]. Pro-inflammatory cytokine IL-1β was found to be linked to neuronal plasticity, which was required for cognitive ability in patients with depression [23]. IL-6 was also observed to be associated with synaptic plasticity, neurogenesis and neuromodulation [24]. TNF-α, regarded as a biomarker of stress, played a primary role in the cerebral innate immunity [24]. In our research, the levels of IL-1β, TNF-α, and IL-6 in BA or fluoxetine (positive control)-treated groups were significantly lower than those in the CUMS group. Chronic stress or depression cause the hyperactivation of the hypothalamic-pituitary-adrenal (HPA) axis, which consequently promote the level of corticosterone. The corticosterone-induced PC12 cells model was commonly used as the in vitro model for depressive animal model [25,26,27]. Our results showed that BA could suppress the generation of pro-inflammatory cytokines including IL-1β, TNF-α, and IL-6 in corticosterone-induced PC12 cells. 

Additionally, it was proved that BA (10, 25, 50, 100 μM) relieved the rotenone-induced death of SH-SY5Y cells in a concentration dependent manner [28]. It was also proved that BA (0.01 and 0.001 mg/mL) did not show significant cytotoxicity in PC12 cells [29]. Our data confirmed that BA (20, 40, 80 μM) treatment had no significant cytotoxicity, but restored the cell viability in corticosterone-induced PC12 cells.

Recently, an inflammatory hypothesis has been proposed for depression development. High-mobility group box 1(HMGB1), belonging to the family of chromatin-associated proteins binding with DNA, is widely expressed in almost all eukaryotic cell nucleus. Substantial evidence displayed that HMGB1 functions as the key trigger for cross-talk between normal cells and inflammatory cells. After the combination between HMGB1 and TLR4, the recruitment and promotion of NF-κB occurred in neuroinflammation [30]. Transcription factor NF-κB is known to activate the transcription of inflammatory cytokines, chemokines and adhesion molecules [31]. Previous studies have illustrated that NF-κB participates in the pathogenesis of inflammation-modulated nerve inflammation, such as depression [32]. The previous study investigated the mechanism of BA on CUMS induced depression by mediating NLRP3 inflammasome in prefrontal cortex of rats [19]. Yu et al. demonstrated the anti-depressive effect BA on CUMS induced rats by modulating AMPA receptor expression [33]. In the present work, together with alterations of pro-inflammatory cytokine levels, our study showed that the expressions of inflammatory-related proteins, such as HMGB1, TLR4 and NF-κB, were increased in the hippocampus of CUMS mice compared with the control group. BA and Flu administrations dramatically down-regulated the HMGB1, TLR4 and p-NF-κBp65 expressions in the hippocampus of CUMS-stimulated mice and corticosterone-induced PC12 cells using Western blot analysis.

In conclusion, the present research demonstrated that BA effectively attenuated CUMS induced depressive behavioral abnormalities in mice. The antidepressant effect mainly resulted from the modulation of the inflammatory condition, which might be associated with the inhibition of the HMGB1/TLR4/NF-κB signaling pathway. 

## 4. Materials and Methods

### 4.1. Reagent

BA, 3-(4,5-Dimethylthiazol-2-yl)-2,5-diphenyl tetrazolium bromide (MTT), corticosterone and dimethyl sulfoxide (DMSO) were obtained from Sigma-Aldrich (Saint Louis, MO, USA). Fluoxetine (Flu) was purchased from Simcere Pharmaceutical group (Nanjing, China). Enzyme-linked immunosorbent assay (ELISA) kits from Nanjing KeyGEN Biotech. CO., LTD were applied for IL-1β, IL-6, TNF-α analyses. (Nanjing, China). High-glucose Dulbecco’s modified Eagle’s medium (DMEM) and fetal bovine serum (FBS) were supplied from GIBCO-BRL (Grand Island, NY, USA). BCA protein quantitation kit was produced by Nanjing Jiancheng Institute of Bioengineering (Nanjing, China). Primary antibodies including anti-HMGB1(#6893), anti-TLR4(#14358), anti-p-NF-κB(#3303), anti-NF-κB(#6956), anti-GAPDH(#2118) were purchased from Cell Signaling Technology (Danvers, MA, USA). Goat Anti-rabbit IgG (H+L) (ab205718) was obtained from Abcam (Cambridge, MA, USA)

### 4.2. Ethics Statement

All the animal experimental procedures were performed strictly according to protocols approved by Medicine Animal Care and Use Committee of Guangdong Medical University (permission number IACUC-20160510). 

### 4.3. Animals

SPF ICR mice (18-22 g) were purchased from Comparative Medicine Center of Yangzhou University. They were housed in standard room with 12 h light-12 h night cycle and 40–60% humidity at 24 ± 2 °C. The mice had free access to standard food and water. All experiments were conducted in accordance with the Provision and General Recommendation of Chinese Experimental Animals Administration Legislation.

### 4.4. Animal Experimental Design

60 mice were randomly divided into five groups as follows (*n* = 12): Control group, model group (CUMS), Flu (20 mg/kg) group, BA (25 mg/kg) group, and BA (50 mg/kg) group. The drug was dissolved in normal saline with DMSO less than 0.1% [*v*/*v*] (vehicle). The dosage of Baicalin and Flu were chosen according to the previous investigation combined with our preliminary experiment results [34,35]. After five days adaption, the CUMS procedure was performed according to a previous publication within minor modification [11] (Table 1). Except for the control group, the mice in the other four groups were exposed to the CUMS challenge for six weeks, from the 21st day, the mice then intragastrically received Flu or BA treatments once daily for three weeks, respectively. Simultaneously, mice in the control group and the CUMS group were orally given vehicle at an equal volume. 1 h after the last drug treatment, the OFT, TST and FST behavior tests were performed within 24 h. There was a 2 h interval between each behavior test. After that, the SPT was carried out and mice were sacrificed. No animals were found dead during the experiment. Finally, the blood was harvested and centrifugated at 2000 g for 10 min and the supernatant was harvested. The brain tissues were immediately snap-frozen and removed on dry ice for dissection of the hippocampus, which were then homogenized with 20% tissue homogenates dissolved in 0.1 M PBS (pH 7.4). The samples were stored at -80 °C for biochemical detection and western blot analysis.

### 4.5. Sucrose Preference Test (SPT)

Sucrose preference test was a measurement to evaluate the responsiveness to positive stimuli. Generally, two bottles of 1% sucrose solution(*w*/*v*) were placed in each cage for 24 h. After the adaption, the mice were suffered for deprivation of water and food. Each mouse was maintained in an individual cage and provided by two bottles supplemented with 150 mL 1% sucrose solution (*w*/*v*) or 150 mL water, respectively. After recording the consumption of sucrose solution and water, the sucrose preference was calculated as [(sucrose consumption)/(water consumption + sucrose consumption)] × 100%. 

### 4.6. Open Field Test (OFT)

The behavior tests were carried out as previous described [36]. In the open field paradigm, mice were placed in the open field apparatus divided into symmetrical sectors. At the beginning, mice were placed in the center of the apparatus. Mice were allowed to freely explore for 6 min. The number of crossings was counted by two trained observers blinded to the experimental group using ANY-maze software 4.99.

### 4.7. Tail Suspension Test (TST)

In this experiment, mice were acoustically and visually isolated [37]. Mice were individually suspended by the end of their tail with adhesive tape (about 50 cm above the floor) in a sound-isolated room. Each mouse was partitioned for a period of 6 min to avoid interference. Finally, the last four minute suspension was recorded as the time of immobility by two trained investigators who were unaware of the strain in a blinded manner using ANY-maze software 4.99. 

### 4.8. Forced Swimming Test (FST)

FST was performed according to previously described methods [38]. Briefly, the mouse was individually placed in a glass cylinder (20 cm height × 14 cm internal diameter) filled with fresh water (10 cm height, 25 ± 2 °C). The immobility time was quantified as the time when mice floated motionless except the necessary movement to keep its head above water. The animals were warmed after the test. The immobility duration was recorded within the last 4 min by two trained observers who were blinded to the experiment using ANY-maze software 4.99. 

### 4.9. Culture of PC12 Cells

PC12 cells were cultured in DMEM plus 10% FBS and 1% antibiotics (penicillin/streptomycin) in a humidified 5% CO_2_–95% air incubator at 37 °C. 

### 4.10. MTT Assay

Cell viability was evaluated by MTT experiment according to manufactory instruction. PC12 cells were supplied from the American Type Culture Collection (ATCC) (Manassas, VI, USA). 5 × 10^4^/mL PC12 cells were seeded on 96-well. 24 h later, the cells were incubated with different final concentrations of BA (10, 20, 40, 80, 160) for 2 h. Then the cells were incubated with corticosterone (800 µM) for 24 h. Next, the cells in each well were exposed to 20 μL of MTT (5 mg/mL, Sigma) solution for 4 h. After the incubation, the culture medium was discarded, and 150 μL DMSO was added. The colorimetric reading was recorded at a test wavelength of 570 nm using a microplate spectrophotometer. Data were estimated as the percentage of the average absorbance to the control group. Cell viability (%) = (A_Treated_/A_Control_) × 100%.

### 4.11. Experimental Design in PC12 Cells

5 × 10^4^/mL PC12 cells were seeded on 96-well or 6 well-plate. 24 h later, the cells were pretreated with BA (20, 40, 80 μM) for 2 h, then the cells were treated with corticosterone (800 µM) for 24 h. The cells and cells supernatant were harvested for pending detection.

### 4.12. Cytokine Assay

The levels of proinflammatory mediators, such as IL-1β, IL-6, TNF-α in serum, hippocampus homogenates and cell culture medium were determined by ELISA kits according to the manufacturer’s instructions. The absorbance values were observed at 450 nm. The results were expressed as picograms per milligram protein.

### 4.13. Western Blot Analysis

The hippocampus samples and PC12 cells were lysed and homogenized in ice-cold radioimmunoprecipitation assay buffer (Beyotime, Nanjing, China) containing 0.1% phenylmethylsulfonyl fluoride. The solution was centrifuged and the supernatant protein concentration was determined using a commercial BCA assay kit (Beyotime, Nanjing). Protein samples were loaded on an 8–12% SDS-polyacrylamide gel electrophoresis, and further transferred onto PVDF membranes (Millipore, MA, USA). The membranes were blocked with 5% skim milk, and then incubated with separate primary antibodies at 4 °C overnight. After washing by Tris-buffered saline-tween 20 (TBST), the PVDF membranes were incubated with secondary antibody at room temperature. The band intensities were visualized by an ECL reagent (KeyGEN Biotechnology, Nanjing, China) and analyzed with a BIO-RAD ChemiDoc XRS system. Densitometric analysis was performed using Image J software (The National Institutes of Health, Bethesda, MD, USA).

### 4.14. Statistical Analysis

All data are expressed as mean ± SEM. Results were analyzed by one way-ANOVA followed by Tukey’s post hoc test with SPSS 17.0 (SPSS Inc., USA). *p* < 0.05 was considered to be a significant difference.

## 5. Conclusion

CUMS-treated mice presented notable depressive-like symptoms, such as decreased sucrose consumption, increased FST and TST immobility time. While BA (25, 50 mg/kg) significantly attenuated these changes. Besides, BA treatment considerably inhibited inflammatory cytokinesl (IL-1β, IL-6, TNF-α) levels in serum, hippocampus homogenate and cell culture medium. Western blot analysis indicated that BA inhibited the expressions of HMGB1, TLR4, and p-NF-κBp65 both in vivo and in vitro. In conclusion, the present study confirmed that BA possessed efficient antidepressant effects on depression, which was possibly related to the inhibition of HMGB1/TLR4/NF-κB pathways.

## Figures and Tables

**Table 1:** The procedure of CUMS.

**Monday**	
9:00	Closed light
11:00	Remove food and water, 20 h cold–wet cage (200 mL water (4 °C)/cage)
**Tuesday**	
9:00	Change dry cage, restore food and water, and 40 min of cage shaking (200 rpm)
9:40	Stop cage shaking, continuous light for 24 h
**Wednesday**	
9:00	Closed light, record animal weight
10:00	24 h of tilted cage (45°), and remove water
**Thursday**	
9:00	Stop tilted cage (45°), restore water, and change to 5 mice/cage
15:00	Change to single cage, remove food
**Friday**	
9:00	Restore food, 40 min of cage shaking (200 rpm)
9:40	Stop cage shaking, 20 h hot–wet cage (200 mL water (45 °C)/cage)
**Saturday**	
9:00	Change dry cage
10:00	24 h of tilted cage (45°), and remove water
**Sunday**	
9:00	Stop tilted cage (45°), restore water, and change to 5 mice/cage
15:00	Change to single cage, continuous light for 20 h

## Abbreviations

BA	Baicalin
CUMS	chronic unpredictable mild stress
DAMP	danger-associated molecular pattern
Flu	fluoxetine
FST	forced swim test
HMGB1	high mobility group box 1 protein
IL-1β	interleukin-1β
NF-κB	nuclear factor kappa B
OFT	open field test
PVDF	polyvinylidene fluoride
TLR4	Toll-like receptor
TNF-α	Tumor Necrosis Factor-α
TST	tail suspension test
TBST	tris-buffered saline-tween
SPT	sucrose preference test
